# Sevoflurane and Desflurane Exposures Following Aneurysmal Subarachnoid Hemorrhage Confer Multifaceted Protection against Delayed Cerebral Ischemia

**DOI:** 10.3390/biomedicines9070820

**Published:** 2021-07-14

**Authors:** Keshav Jayaraman, Meizi Liu, Gregory J. Zipfel, Umeshkumar Athiraman

**Affiliations:** 1 Department of Anesthesiology, Washington University in Saint Louis, Saint Louis, MO 63110, USA; keshav.jayaraman@wustl.edu (K.J.); meizi.l@wustl.edu (M.L.); 2 Department of Neurological Surgery, Washington University in Saint Louis, Saint Louis, MO 63110, USA; zipfelg@wustl.edu

**Keywords:** inhalational anesthesia, sevoflurane, desflurane, conditioning, delayed cerebral ischemia, aneurysmal subarachnoid hemorrhage

## Abstract

Numerous studies have demonstrated the ability of isoflurane conditioning to provide multifaceted protection against aneurysmal subarachnoid hemorrhage (SAH)-associated delayed cerebral ischemia (DCI); however, preclinical studies have not yet examined whether other commonly used inhalational anesthetics in neurological patients such as sevoflurane or desflurane are also protective against SAH-induced neurovascular deficits. We therefore sought to identify the potential for sevoflurane and desflurane conditioning to protect against DCI in an endovascular perforation mouse model of SAH. Neurological function was assessed daily via neuroscore. Large artery vasospasm and microvessel thrombosis were assessed three days after SAH or sham surgery. Four groups were examined: Sham, SAH + room air, SAH + 2% Sevoflurane, and SAH + 6% Desflurane. For the SAH groups, one hour after surgery, mice received 2% sevoflurane, 6% desflurane, or room air for one hour. We found that conditioning with sevoflurane or desflurane attenuated large artery vasospasm, reduced microvessel thrombosis, and improved neurologic function. Given their frequent clinical use and strong safety profile in patients (including those with SAH), these data strongly support further studies to validate these findings in preclinical and clinical studies and to elucidate the mechanisms by which these agents might be acting.

## 1. Introduction

Aneurysmal subarachnoid hemorrhage (SAH) affects approximately 500,000 people/year worldwide [1] and accounts for over 70% of all forms of subarachnoid hemorrhage [2]. Roughly 30% of these patients die and 50% of survivors experience substantial deficits [2,3]. Notably, these poor outcomes are disproportionately influenced by Delayed Cerebral Ischemia (DCI), which is a form of clinical deterioration that manifests multiple days following the initial aneurysmal rupture [4,5]. DCI occurs in approximately 30% of SAH patients [2,6,7,8] and is strongly associated with increased patient mortality [7,8]. While modern pharmacological [9] and neurointerventional [10] treatments offer options for addressing certain aspects of DCI, a robustly effective preventative therapy remains elusive. Comprehensive protection against DCI requires consideration of its diverse neurovascular and neuroelectric underpinnings [4]. Phenomena such as large artery vasospasm, thrombotic events within the microvasculature, and spreading depolarizations have been implicated in the pathophysiology of DCI [4]. Though seemingly disparate, such phenomena may be schematized as diverse manifestations of a shared biological heritage. This understanding of DCI pathogenesis suggests that comprehensive protection can be attained by targeting fundamental biomolecular and cellular roots, rather than individual symptomatic manifestations.

Controlled exposure to a noxious stimulus following an acute injury can be used to induce a variety of protective biomolecular and cellular adaptations [11]. This innovative therapeutic approach, termed conditioning, provides an avenue for exploring comprehensive preventative therapies for DCI [11]. Numerous recent studies, including our own, have demonstrated that isoflurane can offer this form of protection against various DCI-related pathologies [12,13,14,15,16]. The potential for isoflurane conditioning to protect against DCI is noteworthy, given its existing use in the clinical setting. However, it is also important to note that isoflurane is considered less desirable than the other commonly used inhalational anesthetics in the clinic, including sevoflurane and desflurane [17,18,19,20,21,22,23,24,25,26]. Specifically, the solubility of both sevoflurane and desflurane anesthetics in blood is lower, resulting in faster emergence from anesthetic, allowing a more immediate post-operative neurological examination. [21,22,23]. Therefore, findings showing a potential DCI protection for sevoflurane or desflurane may be as, if not more, readily translated into standard-of-care paradigms for SAH care. Additionally, such studies would help inform ongoing comparisons between anesthetic treatments (e.g., inhalational vs. intravenous) and thereby enable optimal anesthetic management for SAH patients.

To the best of our knowledge, no published investigation has prospectively investigated the pathophysiological basis and causative relationships (if any) between sevoflurane or desflurane conditioning and protection against SAH-induced DCI. Through this study, we therefore seek to systematically elucidate whether exposure to sevoflurane or desflurane following SAH can attenuate DCI and improve neurological outcomes.

## 2. Methods

### 2.1. Institutional Approval

These mouse-model experiments were conducted following obtainment of institutional approval and authorization (Institution: Washington University in Saint Louis, Committee: Institutional Animal Care and Use Committee, Protocol: 20180080, Effective: 22 July 2019).

### 2.2. Experimental Design and Animals

**Figure 1:**
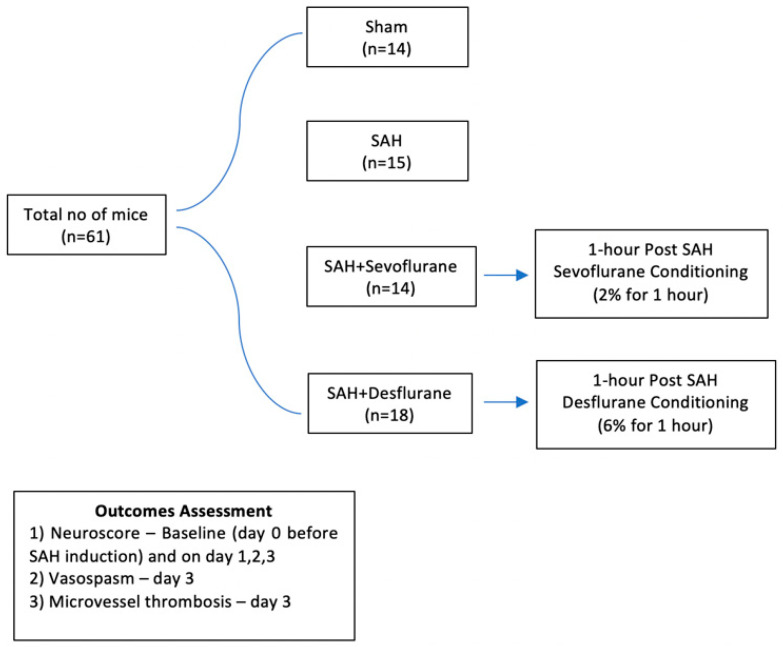
Experimental design of the study. SAH—subarachnoid hemorrhage.

Adult male mice of the C57BL/6J wild-type strain (The Jackson Laboratory, Bar Harbor, ME, USA) were utilized in these investigations. At the time of experimentation, all mice were roughly 12 weeks of age. These prospective experiments were conducted with four treatment groups. Two groups possessed the experimental variables of interest: SAH + Sevoflurane and SAH + Desflurane. Sham and SAH + room air treatment groups were included as procedural and conditioning controls, respectively. Mice were randomly assigned to one of these four experimental treatment groups. Experiments were blinded to the person who conducted the neurological examination. All surgeries were performed by an experienced animal surgeon. For the duration of the experiments and whilst within their home cages, all mice had unrestricted access to food, water, and room air. Figure 1 represents the experimental design of the study.

### 2.3. Murine Model of Aneurysmal Subarachnoid Hemorrhage

Our method for experimentally inducing SAH in mice was adapted from prior work in this domain [12,13,14,15]. In this established model, a suture is inserted into the lumen of the left external carotid artery and progressed cranially until the point at which the left internal carotid artery (L-ICA) branches into the left middle cerebral artery (L-MCA) and left anterior cerebral artery. At the region of this juncture, the suture tip is used to pierce the vessel wall, causing hemorrhage. For control (sham) surgical procedures, the suture tip is similarly maneuvered through the carotid arterial vasculature; however, the tip is withdrawn upon encountering the L-ICA bifurcation aforementioned. SAH induction in the mice was confirmed by the presence of apnea immediately after the suture perforation and by the presence of blood on the ventral surface of brain in SAH animals during the animal sacrifice.

### 2.4. Daily Neurological Examinations

A neurologic examination, referred to as the neuroscore [12,13,14,15], was performed daily for each experimental mouse. The first examination was conducted at baseline prior to experimental surgery (Day 0); the last examination was conducted on the day of sacrifice (Day 3). As detailed in other studies [12,13,14,15], the neuroscore evaluates eight diverse measures of murine neurological function: spontaneously active engagement with surroundings, limb coordination, climbing abilities, balance-related coordination, proprioception, whisker-sensation, vision, and withdrawal response from noxious (tactile) stimuli. Lower numbers on the neuroscore, which ranges from 4 to 24, are taken to indicate greater overall deficits in neurologic function.

### 2.5. Anesthetic Exposure to Sevoflurane or Desflurane

As in other investigations of inhalational anesthetic exposure [12,13,14,15], mice in the anesthetic-conditioning treatment groups were transferred to a small-mammal veterinary anesthesia chamber after being provided one hour of recovery time following SAH surgery. Within this container, mice were exposed for one hour to approximate dosages of either 2% sevoflurane or 6% desflurane. A gas analyzer (Capnomac Ultimata, Datex Ohmeda, Madison, WI, USA) was used to independently monitor chamber anesthesia levels and thereby validate dose accuracy. Exposures were conducted atop a homeothermic blanket, which was in turn linked to a water-recirculating pump that maintained cycling fluid temperatures at 37 °C. This setup was used to ensure viable core temperatures during anesthesia. Dosage levels were chosen to roughly correspond to the one minimum alveolar concentration of these drugs for middle-aged adult humans [27], and in the wild-type mice [28]. The one-hour exposure duration and the timing of application of sevoflurane and desflurane (one hour after SAH) was chosen to ensure consistency with other published studies on isoflurane conditioning. [12,13,14,15].

### 2.6. Imaging of the Left Middle Cerebral Artery

On the 3rd day following SAH (Day 3), mice were sacrificed. A modified version of the procedure detailed by Aum et al. [29] was adapted and implemented to stain the cerebral vasculature while controlling for perfusion time and pressure. Key events within our procedure included: (1) clearance of endogenous blood through vascular perfusion with a solution containing Heparin and Phosphate-Buffered Saline, (2) vascular fixation with a 10% formalin solution, and (3) vascular staining with a solution containing 5-(and-6)-Carboxy-X-rhodamine, succinimidyl ester. Techniques utilized for fluorescence microscopy imaging and analysis of the cerebral vasculature were similar to those described in prior publications [12,13,14,15]. Following extraction of the vessel-casted mouse brain, imaging was performed using a microscope (Nikon Eclipse ME600, Nikon, Tokyo, Japan), fluorescent lighting (X-Cite^TM^ 120PC, EXFO, Quebec City, QC, Canada), and digital camera (Lumenera Infinity 3S, Lumenera Corporation, Ottawa, ON, Canada). Images were acquired and saved using image-processing software (MetaMorph, Molecular Devices, San Jose, CA, USA). The smallest L-MCA diameter within 1 mm of this vessel’s origin was subsequently quantified. The resultant value represents our numerical measure for vasospasm.

### 2.7. Fibrinogen Level Assessment

To quantify the degree of thrombosis within the cerebral microcirculation, fibrinogen levels (measured as percent coverage) were assessed through immunofluorescence. For this procedure, five brains from each of the four experimental treatment groups were arbitrarily selected for fibrinogen quantification. The immunofluorescent staining and imaging procedure utilized is detailed by Liu et al. [15]. To briefly summarize, key steps include: (1) coronal sectioning (50 μM thickness) of brain specimen; (2) incubation in a blocking buffer; (3) incubation in rabbit anti-fibrinogen antibody (1:3000, Abcam, Cambridge, MA, USA); (4) incubation in donkey anti-rabbit secondary antibody (1:2000, Invitrogen Waltham, MA, USA), and (5) imaging (NanoZoomer 2.0-HT, Hamamatsu Photonics K.K, Hamamatsu, Japan).

### 2.8. Statistical Analysis

Statistical analysis was performed on GraphPad Prism 7 (GraphPad Prism 7, GraphPad Software, San Diego, CA, USA). To analyze vasospasm and microvessel thrombosis results, ANOVA (one-way) and a post hoc Student–Newman–Keuls (SNK) statistical test were utilized. For comparison of neuroscore results, a two-factor repeated-measures ANOVA was coupled with the post hoc SNK test. The threshold for statistical significance is a resultant *p*-value less than 0.05.

## 3. Results

### 3.1. Final Sample Sizes and Exclusions

A total of 66 mice were originally obtained for these experiments. Exclusions were made on the basis of either early mortality or inability to obtain clear results: three mice were excluded due to mortality prior to sacrifice and an additional two mice were excluded due to unclear neurovascular imaging results. Therefore, a total of 61 mice were utilized for examining the vasospasm and neurologic function, with the final sample size of Sham (*n* = 14), SAH + room air (*n* = 15), SAH + Sevoflurane (*n* = 14), and SAH + Desflurane (*n* = 18). SAH was noted in the animals that underwent SAH procedure and no blood was seen in the sham group. For microvascular thrombosis assessments, five mouse brains were arbitrarily selected from each of the four aforementioned treatment groups. Final analyzed sample sizes are as follows: Sham (*n* = 4), SAH + room air (*n* = 5), SAH + Sevoflurane (*n* = 5), and SAH + Desflurane (*n* = 5). One exclusion was made for an abnormal outlier in the sham group.

### 3.2. Sevoflurane and Desflurane Conditioning Attenuated SAH-Induced Large Artery Vasospasm in Wild-Type Mice

**Figure 2:**
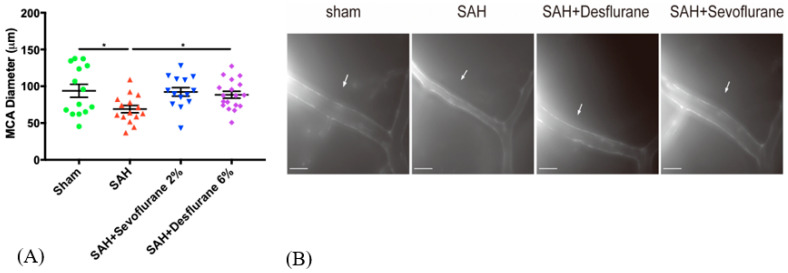
Sevoflurane and desflurane conditioning attenuates SAH-induced vasospasm: Mice were exposed to 2% sevoflurane, 6% desflurane, or room air for one hour, one hour after the SAH or sham surgery. (**A**) Imaging of the L-MCA diameters were performed on day 3. Data are presented as mean ± SEM. A one-way ANOVA and post hoc Student–Newman–Keuls test were used to compare groups: Sham vs. SAH + room air * (*p* < 0.05), SAH + room air vs. SAH + Sevoflurane * (*p* < 0.05), SAH + room air vs. SAH + Desflurane * (*p* < 0.05). (**B**) Representative images of L-MCA. Arrow marks indicate the L-MCA vessel. Scale corresponds to 500 μM.

Upon imaging, as expected, the SAH group had significant vasospasm compared to the sham group. (*p* < 0.05). Both the sevoflurane and desflurane treatment groups were found to have significantly less vasospasm than the SAH group (*p* < 0.05, Figure 2A,B) and no significant difference was noted between the two anesthetics with respect to the vasospasm protection.

### 3.3. Sevoflurane and Desflurane Conditioning Attenuated SAH-Induced Microvessel Thrombosis in Wild-Type Mice

**Figure 3:**
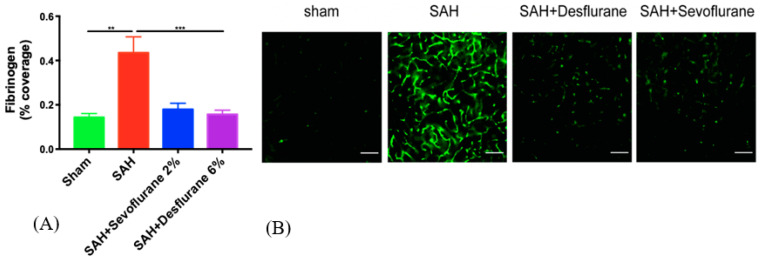
Sevoflurane and desflurane conditioning attenuates SAH-induced microvessel thrombosis: Mice were exposed to 2% sevoflurane, 6% desflurane, or room air for one hour, one hour after the SAH or sham surgery. (**A**) Fibrinogen percent coverage (%) was examined on day 3. Data are presented as mean ± SEM. A one-way ANOVA and post-hoc Student–Newman–Keuls test were used to compare groups: Sham vs. SAH + room air ** (*p* < 0.01), SAH + room air vs. SAH + Sevoflurane *** (*p* < 0.001), SAH + room air vs. SAH + Desflurane *** (*p* < 0.001). (**B**) Representative images—fibrinogen immunofluorescence assay. Scale corresponds to 100 μM.

The SAH + room air group showed more extensive microvascular thrombosis than the sham group (*p* < 0.05) and exposure to sevoflurane and desflurane significantly attenuated microvessel thrombosis (*p* < 0.05, Figure 3A,B). No difference in protection was noted between both the anesthetics.

### 3.4. Sevoflurane and Desflurane Conditioning Improved Neurological Outcomes after SAH in Wild-Type Mice

**Figure 4:**
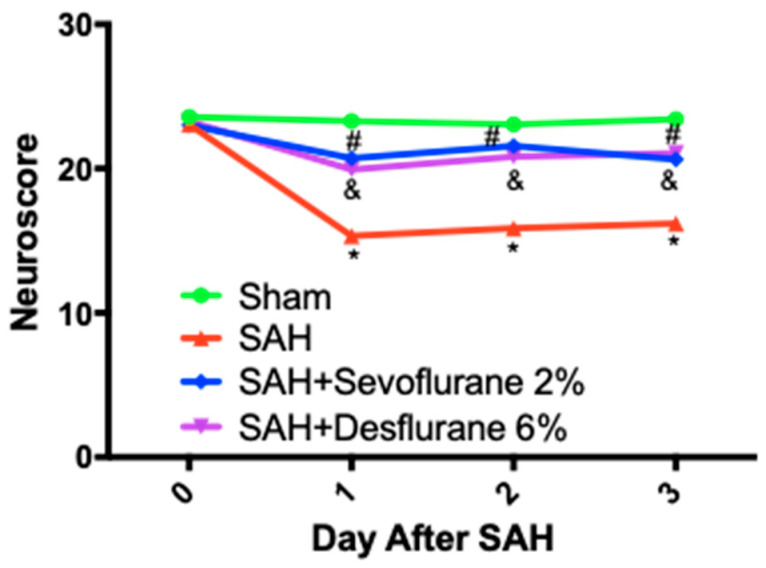
Sevoflurane and desflurane conditioning improves neurologic outcomes after SAH: mice were exposed to 2% sevoflurane, 6% desflurane, or room air for one hour, one hour after the SAH or sham surgery. Higher neuroscores indicate better neurological function. Day 0 neuroscores represent baseline status; mice were sacrificed on Day 3. A two-factor repeated-measures ANOVA and a post hoc Student–Newman–Keuls test were used to compare treatment groups: Sham vs. SAH + room air * (*p* < 0.05), SAH + room air vs. SAH + Sevoflurane ^#^ (*p* < 0.05), SAH + room air vs. SAH + Desflurane ^&^ (*p* < 0.05).

As expected, the SAH + room air group had significantly worse neurologic function than the sham group (*p* < 0.05). Both sevoflurane and desflurane groups had improved neurologic function compared to the SAH + room air group (*p* < 0.05), and no significant difference was noted between both anesthetics. (Figure 4).

## 4. Discussion

The key findings in our study are as follows: (1) both sevoflurane and desflurane conditioning provided strong protection against large artery vasospasm and microvessel thrombosis after SAH; and (2) sevoflurane and desflurane conditioning improved short-term neurological outcomes after SAH. These findings are important for three key reasons: (1) they provide additional evidence to the growing body of experimental literature indicating inhalational anesthetics as a conditioning agent class provide robust neurovascular protection in SAH; [12,13,14,15,16] (2) they show that the much more commonly used inhalational anesthetics, sevoflurane and desflurane, provide potent and multifaceted protection against DCI in SAH, very similar to that previously reported with isoflurane conditioning; [12,13,14,15,16] and (3) they provide preclinical support for the recently reported protective effect of sevoflurane and desflurane against angiographic vasospasm and DCI in SAH patients [30,31,32]. Given the pharmacologic advantages of sevoflurane and desflurane over isoflurane (e.g., the blood–gas and the oil–gas partition coefficient of isoflurane is significantly larger than that of sevoflurane and desflurane [25,26]—both of which lead to prolonged clinical emergence [19,20,21] from general anesthesia that can confound post-procedure neurological assessment), the very high usage rate of sevoflurane and desflurane in neurosurgical patients, and the proven safety track record of desflurane and sevoflurane in a wide variety of patients, the results from our study have relevant and meaningful implications for the care of SAH patients that deserve further investigation.

### 4.1. Sevoflurane Conditioning in SAH

To the best of our knowledge, there are no preclinical studies in the published literature that examine whether sevoflurane conditioning is capable of providing protection against DCI. The results of our study showing that a clinically relevant dose of sevoflurane affords protection against vasospasm and microvessel thrombosis and improves neurologic function is novel and further supports the use of inhalational anesthetic conditioning approaches in DCI prevention. It is important to note that sevoflurane may have numerous additional clinical advantages as an inhalational anesthetic. First, sevoflurane has been shown to cause less respiratory irritation than either of the other inhalational anesthetics being discussed [24]. Second, sevoflurane may possess benefits over isoflurane with regards to blood flow and vascular autoregulation [22]. Third, recent rodent-model studies suggest that sevoflurane also mitigates another form of secondary brain injury caused by SAH, namely early brain injury (EBI) [33,34,35]. Sorar et al. noted that one-hour exposures to either 1.5% or 3% sevoflurane initiated one hour after SAH in a mouse endovascular perforation model protected against cerebral edema and neuronal apoptosis and improved neurobehavioral outcomes at 24 h post SAH [33]. Altay et al. found that 3% sevoflurane and 2% isoflurane offered comparable degrees of prevention against neuronal apoptosis and cerebral edema, leading to improved neurological function [34]. Both studies suggested that the EBI protection by inhalational anesthetics were mediated through the sphingosine kinase pathway. A more recent study by Beck-Schimmer et al. found that sevoflurane sedation for 4 h initiated 15 min post SAH using a rat endovascular perforation model attenuated cerebral edema by stabilizing the adherens junction [35]. Future preclinical studies should aim to elucidate the mechanism and therapeutic window of sevoflurane conditioning. Additionally, it remains to be determined whether sevoflurane’s protective abilities extend to long-term neurologic outcomes and to other features of DCI pathophysiology.

### 4.2. Desflurane Conditioning in SAH

Several clinical studies, including our own, have suggested that desflurane exposure during aneurysm repair in SAH patients is associated with reduced incidence of angiographic vasospasm and DCI [30,31,36]. However, to date, no published preclinical studies have prospectively investigated this relationship. Our novel preclinical findings showing desflurane’s ability to afford strong protection against SAH-induced DCI provide further salient insights that support previous clinical data. Further studies are warranted to investigate the therapeutic window, underlying mechanisms, and long-term outcomes of desflurane conditioning. Moreover, it is imperative to investigate whether, like sevoflurane and isoflurane [34], desflurane might also provide protection against EBI.

### 4.3. Anesthetic Conditioning and Microvessel Thrombosis

Multiple neurovascular features have been implicated in DCI pathophysiology [4,5]. Relative to large-vessel vasospasm, the phenomenon of microvascular thrombus formation has been a recent addition to our understanding of DCI [37]. While a protective role against microvessel thrombosis in a DCI-specific setting has been proposed for isoflurane conditioning [12,15], our findings with sevoflurane and desflurane provide novel evidence that inhalational anesthetics as a class might be able to protect against DCI-associated microvessel thrombosis. While multiple features of the thrombus-forming cascade have been identified and proposed [37], it remains unclear as to how these inhalational anesthetics might be exerting their effects on the cerebral microvasculature. Further preclinical studies aiming to elucidate how the different anesthetic agents are acting at the molecular level will enable the discovery of novel targets for DCI therapeutics and enable a more practical understanding of microvessel thrombosis.

## 5. Conclusions

Our data support sevoflurane and desflurane as conditioning agents capable of offering robust DCI protection after SAH. Given the existing use of these agents in contemporary anesthetic practice and the necessity for multifaceted protection in SAH, our findings possess both translational potential and clinical impact. Investigations aiming to validate and further elucidate these roles are warranted to empower incorporation into standard-of-care DCI prevention paradigms.

## Data Availability

All data are present within the manuscript or available by request to corresponding author, U.A. (uathira@wustl.edu).

## Abbreviations

SAH	Subarachnoid hemorrhage.
DCI	Delayed cerebral ischemia.
EBI	Early brain injury.
MCA	Middle cerebral artery.
ICA	Internal carotid artery.
ANOVA	Analysis of variance.
SNK	Student–Newman–Keuls.
